# Current preoperative strategies applied in the Dutch bariatric centers: A national survey

**DOI:** 10.1111/cob.12461

**Published:** 2021-05-24

**Authors:** Aniek M. Kolen, Marleen M. Romeijn, Daniëlle D. B. Holthuijsen, Loes Janssen, Jan Willem M. Greve, Wouter K. G. Leclercq, François M. H. van Dielen

**Affiliations:** ^1^ Department of Surgery Máxima Medical Center Veldhoven The Netherlands; ^2^ Faculty of Health, Medicine and Life Sciences Maastricht University Maastricht The Netherlands; ^3^ Research School NUTRIM, Department of Surgery Maastricht University Medical Center Maastricht The Netherlands; ^4^ Department of Surgery Zuyderland Medical Center Heerlen The Netherlands

**Keywords:** bariatric surgery, diet, physical activity, preoperative care, supplements, weight loss

## Abstract

There is no consensus about the optimal management of patients undergoing bariatric surgery. This study aimed to identify current weight loss goals prior to bariatric surgery, as well as aimed to explore preoperative strategies related to diet, nutritional supplements and physical activity. An online survey was distributed among bariatric surgeons and dietitians in all 18 Dutch bariatric centers. This survey included the following four domains: weight loss, diet, nutritional supplements and physical activity. For the analyses one answer per center was used, either the most common answer or the answer given by the most expert responder. All 18 centers reported at least one response. Preoperative weight loss was requested in 28% of the centers, whereas 61% desired a stable weight or weight loss, and 11% had no requests. A preoperative diet was routinely recommended in 78% of the centers and on indication (ie, depending on baseline weight and/or comorbidity status) in 22%. The most frequently prescribed diet was a low‐energy diet (800‐1500 kcal/day) in 44% of the centers. Nutritional supplements were recommended in 78% of the centers. Physical activity with low intensity was recommended in 83% of the centers, while physical exercise training with mid‐ to high‐intensity was recommended in 72%. Inconsistent responses within centers were observed in 56% of the questions. The current bariatric practice within the Netherlands shows high variability and inconsistencies in preoperative management. Consensus‐building and standardization of strategies should be promoted in the future.

**What is already known about this subject?**

- There is no consensus about the optimal management, in terms of dietary and exercise regimes, in patients undergoing bariatric surgery.
- An energy‐restricted diet is often prescribed before bariatric surgery to reduce weight and liver volume.

**What this study adds?**

- Large varieties in preoperative management of all 18 Dutch bariatric centers were observed, as well as inconsistencies in responses within centers.
- This study highlights the need for consensus building in the management of patients undergoing bariatric surgery.

**Abbreviations**
ASMBS: American Society for Metabolic and Bariatric Surgery
DSMBS: Dutch Society of Metabolic and Bariatric Surgery
LED: low‐energy diet
NDBC: Netwerk Diëtisten Bariatrische Chirurgie (in English: Society of Dietitians in Bariatric Surgery)
VLED: very low‐energy diet

## 1 INTRODUCTION

Bariatric surgery is considered the most effective treatment for severe obesity.^1^, ^2^ Over the past years, approximately 11 500 bariatric procedures have been performed annually in the Netherlands.^3^ These procedures are considered safe as only 2.8% of the patients develops a major complication within 30 days after primary surgery.^4^ Due to an altered anatomy in patients with morbid obesity, bariatric surgery can be technically challenging. These challenges are related to abdominal wall thickness, increased visceral adiposity and the presence of an enlarged liver reducing intra‐abdominal space.^4^, ^5^ This may increase the difficulty of the surgical procedure. In order to overcome these challenges, it is conducive that a patients' liver volume and weight are preoperatively lowered.^5^, ^6^, ^7^

Key aspects of preoperative strategies can be listed into energy‐restricted diets and physical activity. In terms of energy‐restricted diets, both very low‐energy diets (VLED, <800 kcal/day) and low‐energy diets (LED, 800‐1500 kcal/day) are considered to be effective.^5^, ^6^, ^7^ Systematic reviews reported a reduction in liver size (5%‐20% VLED; 12‐27% LED),^5^, ^7^, ^8^ intrahepatic fat (43% VLED; 40%‐51% LED)^7^, ^9^, ^10^ and body weight (2.8‐14.8 kg VLED; 5.4‐23.6 kg LED).^5^, ^7^, ^8^ Regardless of the selected dietary strategy it is recommended to assess and, if necessary, supplement micronutrients (eg, iron, zinc, calcium, folic acid, vitamin D and B12) as this may improve overall health.^11^ In terms of physical activity, a variety of exercise programs have shown to be beneficial in the preoperative phase. These programs last 1 to 24 weeks and consist of at least partially supervised trainings, with an intensity of 65% VO2 max and 55% to 85% peak heart rate.^12^ These exercise programs reduce weight (4.1‐5.0 kg) with possible maintenance of lean body mass, as well as improve cardiometabolic risk factors and physical fitness.^11^, ^12^ The exact effect of these exercise programs on liver volume is unknown, nonetheless it has shown to be effective in improving fatty liver disease.^13^

According to the American Society for Metabolic and Bariatric Surgery (ASMBS) in 2016, there is no level A evidence about the most optimal type of preoperative weight loss program (ie, dietary or exercise strategies) and neither about the content and duration of this program.^14^ A high variability in preoperative work up has since then been described in multiple countries,^15^, ^16^, ^17^ yet it is unknown if and how this applies in the Dutch bariatric centers. The primary aim of this study was to identify variations in current weight loss goals prior to bariatric surgery.

## 2 MATERIALS AND METHODS

### 2.1 Study population

In the Netherlands, bariatric surgery is performed in 18 centers and these centers can only be certified if at least 200 bariatric procedures are performed each year.^4^ Centers performing bariatric surgery can be described as non‐academic teaching hospitals and non‐academic non‐teaching hospitals. A survey study was performed among professionals in all Dutch bariatric centers. Bariatric surgeons, surgical residents, physician assistants and nurse practitioners of the Dutch Society of Metabolic and Bariatric Surgery (DSMBS) were invited to participate in an online survey. Dietitians specialized in bariatric care affiliated with the Society of Dietitians in Bariatric Surgery (Network Dietitians Bariatric Surgery, NDBC) were invited as well. Both societies contacted their members by email in April/May 2020. In this email, the content of the study and a weblink to the survey were provided. Centers with no dietitians associated to the NDBC were contacted separately by email. Only surveys that were completed for >80% were included in this study. We aimed to include at least one respondent, either a bariatric surgeon or a dietitian, per center.

### 2.2 Study parameters

The primary study outcome was the variability in preoperative strategies related to weight loss goals in the 18 Dutch bariatric centers. The secondary outcome was the applied strategies in terms of diet, nutritional supplements and physical activity. Dietary advice was listed into composition‐, duration‐ and consistency of the diet, as well as the number of eating moments per day and fluid intake. Nutritional supplements were listed into multivitamin, calcium and vitamin D, protein and probiotics. Physical activity was classified into low‐intense activity and moderate‐ to high‐intense activity (ie, exercise training). If possible, information about the type, frequency, duration and facilitated supervision of physical activity was collected. Other outcomes involved substantiation and experience with the preoperative strategy, as well as the level of inconsistency in responses within a center.

### 2.3 Survey

A web‐based survey was designed by two researchers (A. K., M. R.), one bariatric surgeon (F. D.) and one dietitian specialized in bariatric care. The survey was developed based on prior studies^15^, ^16^, ^17^ and was administered using Qualtrics electronic survey software.^18^ Survey replies were registered anonymously; however, the type of center and profession were asked. The survey consisted of 60 questions, but the actual survey length could vary between 9 and 60 questions since display and skip logic was included to benefit survey flow. The survey contained the following four domains: preoperative weight loss (2‐3 questions), diet (1‐21 questions), supplement use (1‐24 questions) and physical activity (2‐9 questions). Questions were mainly designed as multiple‐choice (28 questions). Open questions or text entry boxes were inserted to obtain additional information (29 open questions, 8 text entry boxes). Other question designs included a slider (2 questions) and “pick and rank” order question (2 questions). The survey was conducted in Dutch. In order to increase international understanding of this article, the survey was translated to English (Table S1).

### 2.4 Data analysis

The Statistical Package for the Social Sciences for Windows (version 22.0; IBM SPSS Inc, Chicago, Illinois) was used for descriptive data analysis.^19^ Categorical data were expressed in numbers and percentages. Continuous data were expressed in mean (SD, range) or median (range) depending on data distribution. Names of the centers were removed and substituted by a random code between 1 and 18.^20^ To obtain one protocol per center, answers from different respondents within the same center were combined by two researchers (A. K., D. H.) and crosschecked by a third researcher (M. R.). In case of nominal variables (yes/no/I do not know), the answer “I do not know” was neglected and the most frequent response was used for the combined protocol. In case of continuous variables, a mean was calculated and used as a final result. In case of ordinal variables, one ranking was made based on the most chosen answer at the most frequent position. When considering multiple‐choice questions, the answers that were provided by at least half of the respondents were used for the combined protocol. By equal responses, the bariatric surgeon's answer was leading in weight goal questions and the dietitian's answer in diet and nutritional supplements questions. The level of inconsistency was identified for all four domains and an overall score was calculated. If at least one response was different compared to other responses within a center, this was classified as inconsistent. A median of inconsistency was calculated by expressing the amount of different responses as a percentage to the total amount of responses.

## 3 RESULTS

**FIGURE 1:**
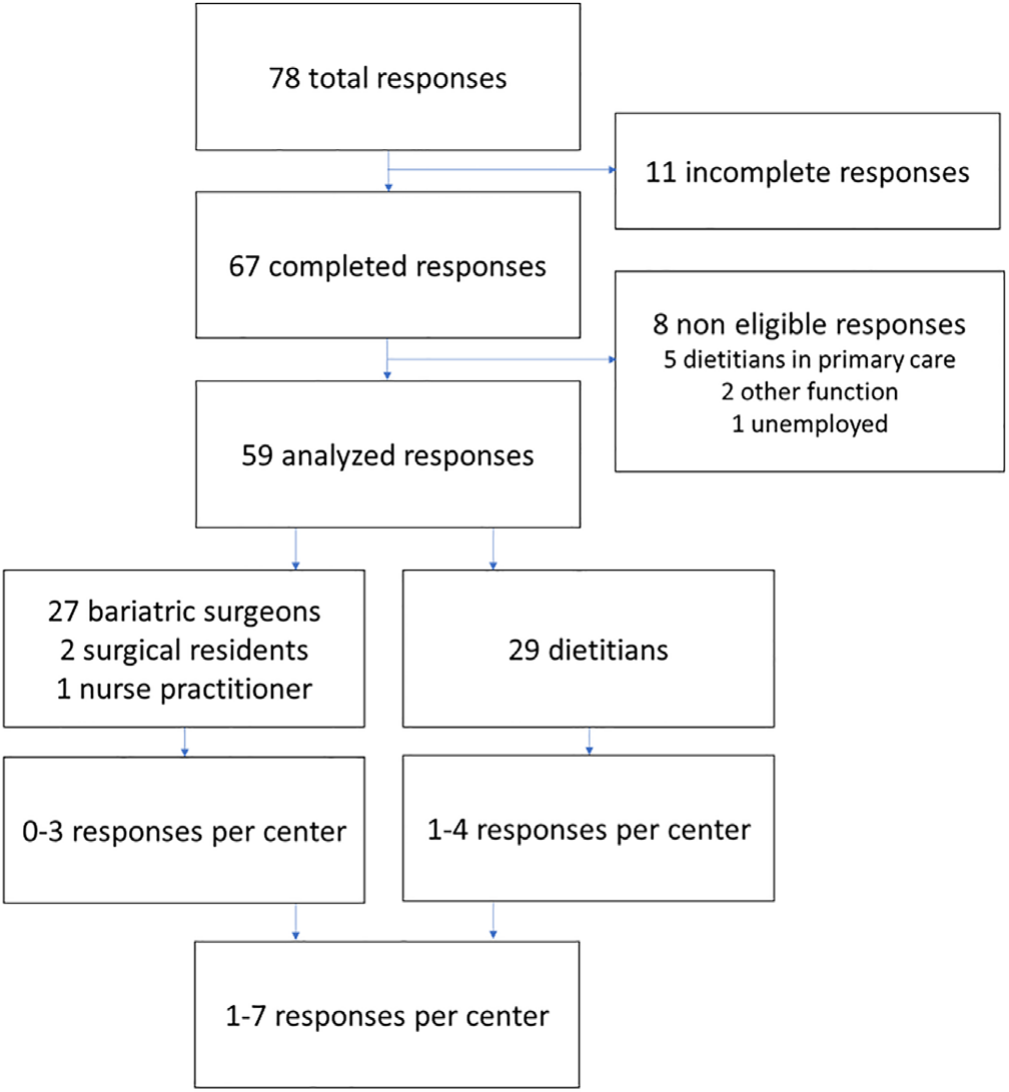
Flowchart of the study inclusion

Within an eight‐week inclusion period, bariatric surgeons and dietitians from all 18 bariatric centers in the Netherlands responded to the survey. There was one center with a response from a bariatric surgeon that did not reach the 80% completion rate, therefore this response was excluded. Yet, this center was included in the analysis because the dietitian responded adequately. From the 78 responses, 59 responses were included in the analysis (Figure 1). Main reasons for exclusion were respondents working in a setting other than in a bariatric center (n = 5), respondents working in a field other than bariatric surgery (n = 2) and respondents who were currently unemployed (n = 1). In 11 centers the overall preoperative protocol was implemented for over 5 years, in 4 centers more than 3 years, in 2 centers 1 year and in 1 center less than 1 year.

### 3.1 Weight loss goal

**FIGURE 2:**
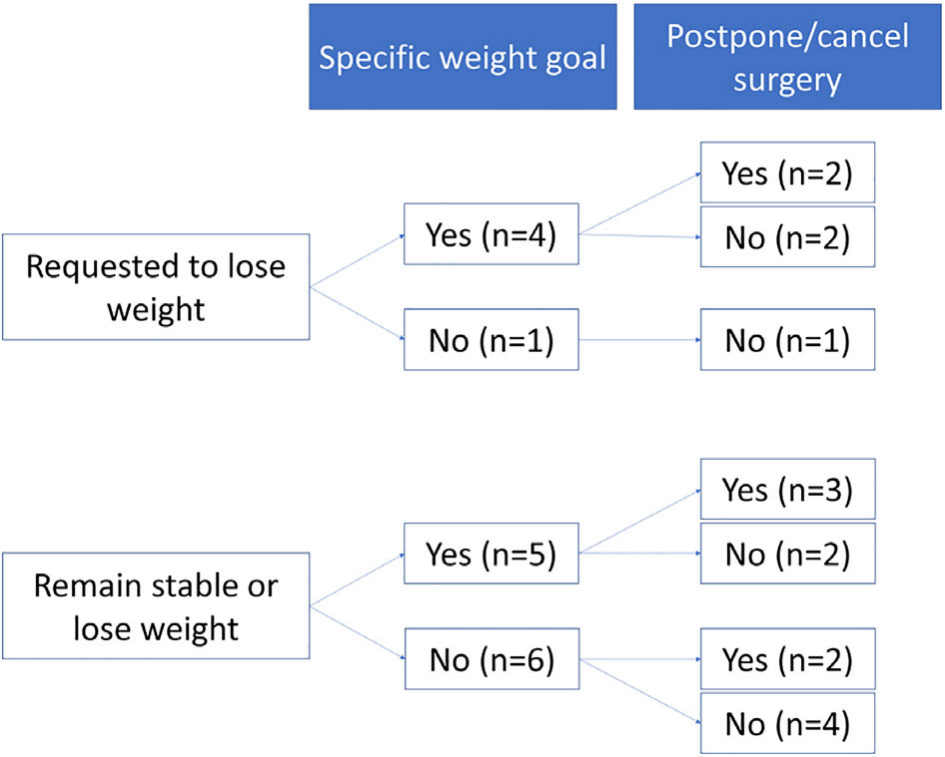
Flowchart of weight loss goals and postponement of surgery in 16 bariatric centers

Preoperative weight loss was requested in 5 (28%) centers, while patients had to remain stable on their weight or lose weight in 11 (61%) centers. Two (11%) centers did not set any weight loss goals (Table 1). In case centers requested a specific weight loss (44%, n = 8), it was usually between 3 to 10 kg and/or 5% to 10% of total weight loss. In 7 (39%) centers, surgery would be cancelled or postponed if the desired weight was not obtained (Figure 2).

**TABLE 1:** Overview of preoperative recommendations given by 18 bariatric centers

Overview of preoperative recommendations	Number of centers	Percentage
Weight loss		
Lose weight	5	28%
Remain stable or lose weight	11	61%
Weight does not matter, may even gain weight	2	11%
Dietary prescription		
Yes	14	78%
No	0	0%
On indication	4	22%
Use of nutritional supplements		
Yes	14	78%
No	3	17%
Unknown	1	6%
Increase of physical activity		
Yes	15	83%
No	3	17%
Unknown	0	0%

### 3.2 Dietary recommendations

**FIGURE 3:**
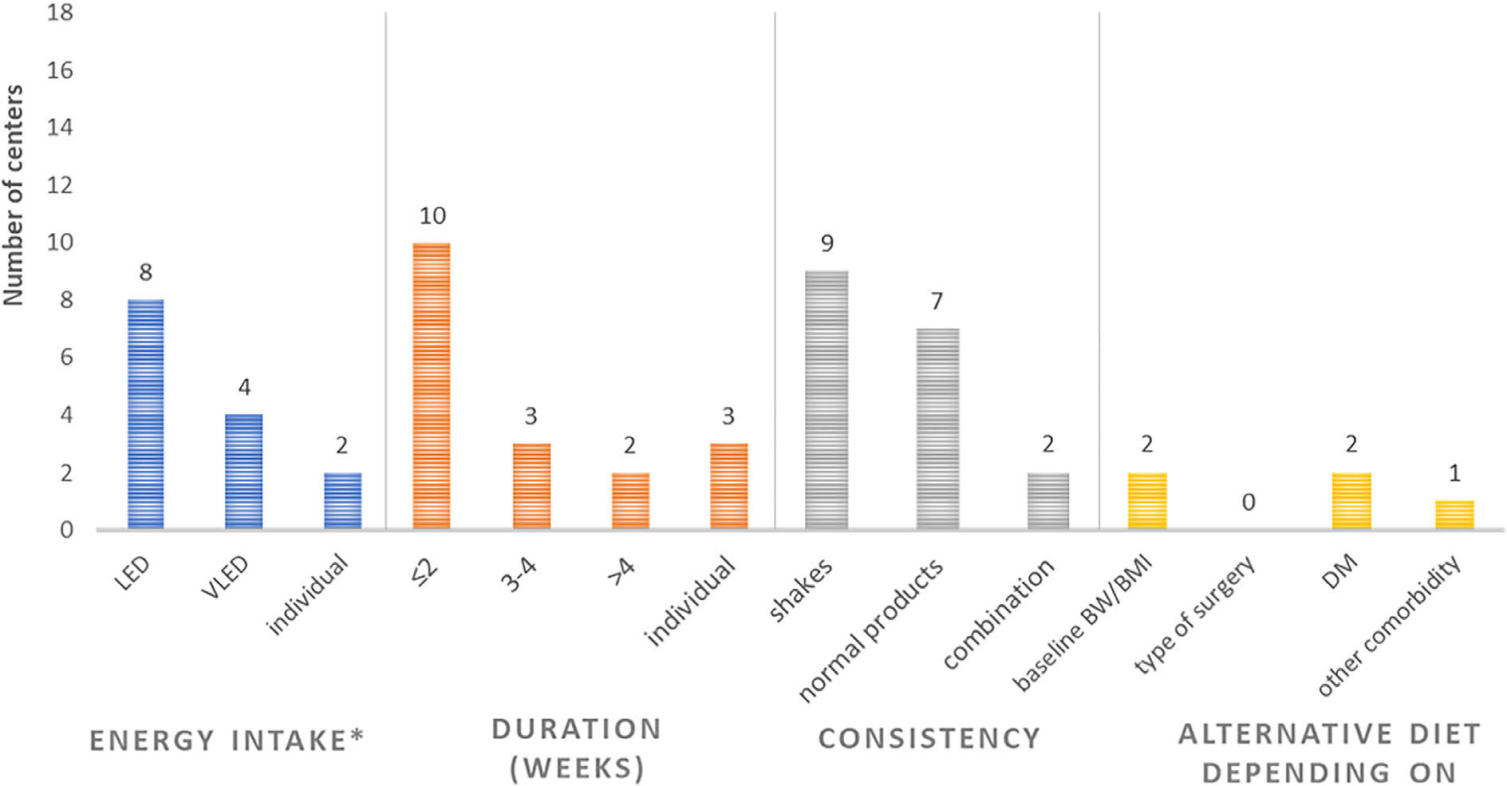
Characteristics of the diets, recommended in the preoperative phase by 18 bariatric centers. *In four centers, the amount of kcal/day was unknown. BMI, body mass index; BW, body weight; DM, diabetes mellitus; LED, low energy diet; VLED, very low energy diet

A specific diet was routinely recommended in 14 (78%) centers, while in 4 (22%) centers this was done only on indication (Table 1). The recommended diet contained between 500 and 1500 kcal per day in 12 centers, with 8 (44%) of the centers recommending an LED and 4 (22%) a VLED (Figure 3). In 4 centers, the amount of kcal/day was unknown, while in 2 centers the energy intake of the corresponding diet was tailored to the individual patient. The duration of the diet ranged between 1.5 and 7.5 weeks, with a median of 2 weeks. In 2 centers, the duration of the diet depended on baseline body mass index. The most important goal of the recommended diet was liver volume reduction (60%, n = 11), followed by reduction of complications (17%, n = 3) and preparation of patients for post‐surgery eating habits (11%, n = 2; Figure S1).

All 11 centers that recommended full or partial liquid meal replacements allowed patients to consume regular products next to the recommended diet. These products included raw vegetables in 11 (100%) centers, clear soups in 10 (91%) centers, steamed/boiled vegetables in 6 (55%) centers and dairy products in 5 (46%) centers. Eight centers (44%) recommended a protein intake between 51 and 95 g per day, carbohydrate intake between 30 and 127 g per day and fat intake between 3 and 28 g per day; of the other centers, macronutrient composition of the diet was unknown. Recommendations regarding fluid intake were given in 16 centers and ranged between 1.5 and 4.0 liters per day, with most of the centers recommending patients to consume 1.5 to 2.0 liters per day. The number of eating moments ranged between four and six times per day. Compliance with the recommended diet was estimated between 75% and 100% by 13 centers. The dietary protocol was based on clinical experience in 16 (89%) centers, on scientific evidence in 10 (56%) centers and on guidelines in 9 (50%) centers.

### 3.3 Nutritional supplement recommendations

Nutritional supplements were recommended in 14 (78%) centers, while 3 (17%) centers did not recommend these supplements (Table 1). Multivitamin supplements were routinely recommended in 10 (59%) centers, while 2 (12%) centers recommended multivitamin supplements only on indication (eg, deficiency). The multivitamin supplementation was generally recommended between 2 and 4 weeks before surgery and the type and dose depended on the type of surgery. As shown in Table 2, calcium and vitamin D supplementation was recommended routinely by 2 (12%) centers and most of the centers (82%, n = 14) did not recommend protein supplementation.

**TABLE 2:** Overview of nutritional supplements recommended in the preoperative phase by 17 bariatric centers

Overview of nutritional supplements	Number of centers	Percentage
Multivitamin		
Yes	10	59%
No	5	29%
On indication	2	12%
Calcium and vitamin D		
Yes	2	12%
No	5	29%
On indication	10	59%
Protein		
Yes	0	0%
No	14	82%
On indication	3	18%
Probiotic		
Yes	0	0%
No	16	94%
On indication	1	6%

### 3.4 Physical activity recommendations

Any form of physical activity (ie, low intensity) was recommended in 15 (83%) centers (Table 1), while actual physical exercise training (moderate‐ to high‐intensity) was recommended in 13 (72%) centers. Three centers (17%) facilitated supervised physical exercise training to all patients, while 2 centers (11%) facilitated this only on indication (eg, patients with low aerobic fitness). Four centers recommended patients to adhere to the Dutch Physical Activity Guidelines.^21^ The type, frequency and duration of physical activity being recommended was frequently unknown. The main goals of the centers that recommended physical activity were behavioural change (63%, n = 10) and improving overall physical fitness (38%, n = 6).

### 3.5 Inconsistencies within centers

**FIGURE 4:**
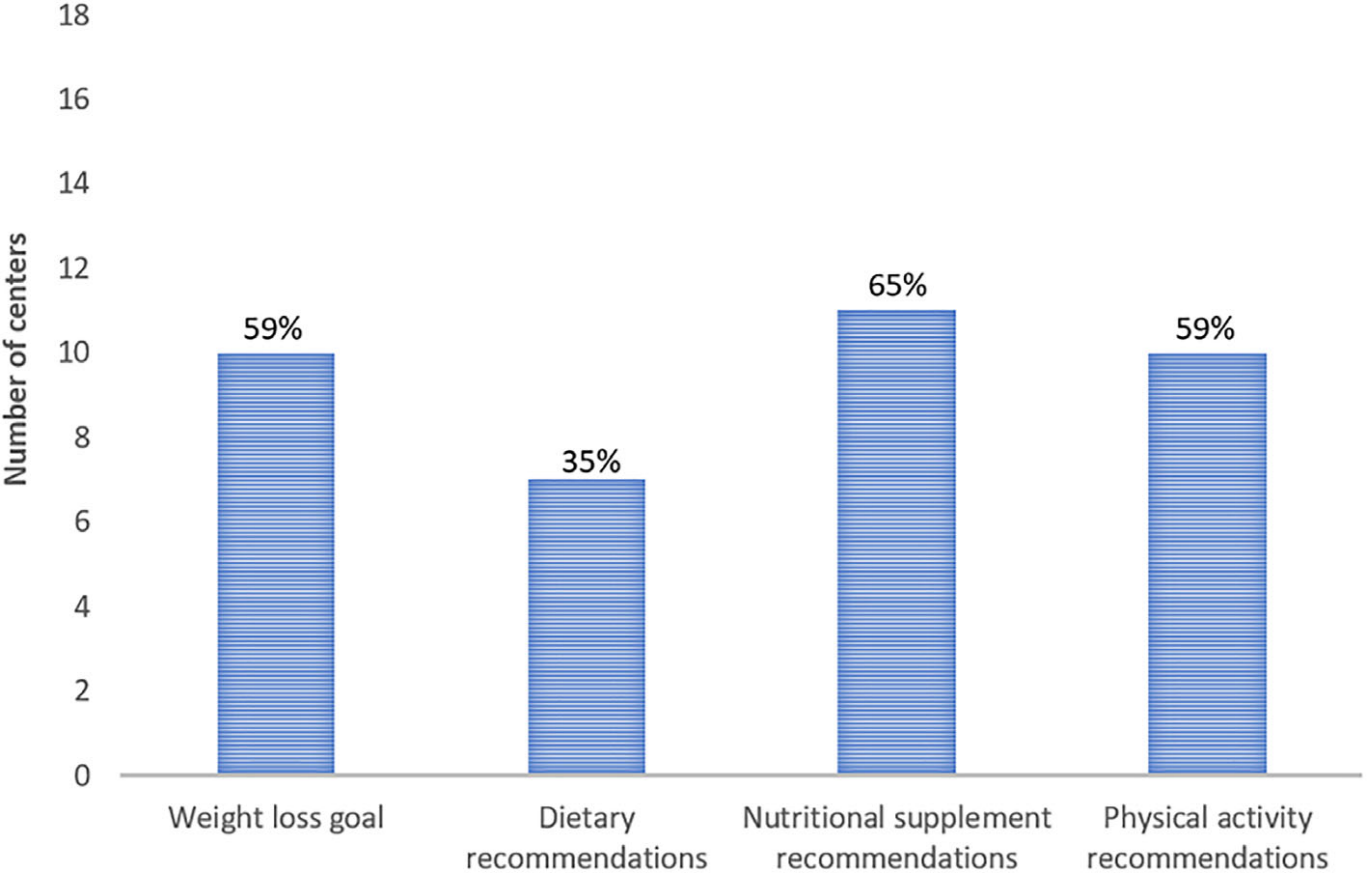
Overview of inconsistent answers from respondents based on the four domains

Over half of the questions (56%), reflecting the four domains, were answered inconsistently by respondents within the same center. The greatest inconsistency was found in the domain of nutritional supplements (65%) followed by weight loss goals (59%) and physical activity (59%) (Figure 4). Respondents of only 1 center provided no inconsistent answers, while in 12 (71%) centers the respondents answered 50% to 100% of the questions inconsistent. The median of inconsistent answers within a center was 25%.

## 4 DISCUSSION

This study aimed to identify current weight loss goals prior to bariatric surgery in the Dutch bariatric practices, as well as to explore current preoperative strategies related to diet, nutritional supplement and physical activity. The most obvious finding that emerged from this study was the large variation in applied strategies and inconsistencies in responses within centers. These inconsistencies were described in 56% of all questions and covered all domains. This implicates that centers need to collaborate in multidisciplinary teams in order to align their preoperative protocols.

With respect to preoperative weight loss, the majority (72%) of the centers did not request weight loss. The absolute necessity for preoperative weight loss is arguable and based on recently updated Dutch guidelines, surgery should be performed irrespective of preoperative weight loss.^22^ Preoperative weight loss has been associated with a decreased liver volume and a decreased surgical complexity, but inconsistent data has been found for short‐term outcomes like complication rate and hospital stay.^14^, ^23^ Furthermore, there is no evidence that long‐term outcomes are improved by better preoperative weight loss.^14^ These findings likely explain the variety found in weight loss goals.

Preoperative dietary regimes greatly differ per country.^15^, ^16^, ^17^ This study identified that particularly in the Netherlands, an LED was the most commonly prescribed diet while for example, Australia seemed to prefer VLEDs.^17^ Both diets have shown to be effective in reducing liver volume^5^, ^7^; however, an LED might be advantageous as it avoids unnecessary energy restriction and may improve dietary compliance. This study identified that the median duration of the diet was 2 weeks. This duration seems to be sufficient as researchers found that 80% to 100% of liver volume reduction occurred within the first 2 weeks of dieting.^9^, ^24^

Centers reported limited and diverse recommendations regarding nutritional supplements, whilst there is a proven high prevalence of micronutrient deficiencies in bariatric candidates.^25^, ^26^ These deficiencies negatively affect the patient's health as it may result in anaemia, peripheral neuropathy, osteoporosis and bleeding disorders.^25^, ^26^ Despite these risks, this study as well as prior studies reported that nutritional supplementation is frequently omitted in the preoperative phase.^15^, ^16^ In this study, calcium and vitamin D supplementation was not recommended in five centers (29%). This does not enterily match the position of the ASMBS recommending to perform a nutritional assessment in all patients prior to bariatric surgery, and to anticipate on any deficiencies.^27^ The Dutch guideline has not taken a position on this point^22^ making it plausible that preoperative nutritional assessments and subsequent regimes have varied across centers.

It is well known that physical activity is beneficial for improving overall fitness and health. In the field of bariatric surgery, structured preoperative physical exercise training including aerobic and strength training for 3 times a week for 12 weeks, is associated with a greater decrease in body mass index postoperatively, and is effective in increasing physical fitness 1 year after surgery.^28^ The current survey showed that 83% of the centers recommended patients to increase their low‐intense physical activities, while only 72% recommended patients to increase their moderate‐ to high‐intense activities. An implication of these findings is that more centers recognize the advantages of moderate‐ to high‐intense activities preoperatively and recommend patients to perform these activities.

Since this survey used a non‐validated questionnaire, the questions could be interpreted slightly different by the respondents than anticipated by the researchers. Moreover, the assessment of physical activity was limited since physical therapists were not invited as respondents. Notwithstanding these limitations, the study had a response from every Dutch bariatric center and offers valuable insights into the commonly used preoperative strategies. It would be interesting to understand the impact of the different preoperative strategies on clinical outcomes like complications, weight loss and comorbidity resolution. This information was not available in this study, but would be recommended in future research.

In conclusion, this study indicates that there is a high variability in preoperative care in the Dutch bariatric centers and reveals large inconsistencies between respondents within the same center. Alignment of local protocols should be a priority for multidisciplinary teams. Well‐designed studies are warranted as they can contribute to the development of (intern)national guidelines and may build upon consensus about the best preoperative strategy.

## CONFLICT OF INTEREST

The authors declare no potential conflict of interest.

## Supporting information

**AppendixS1**: Supporting information.
