# Supplementary material for: Current preoperative strategies applied in the Dutch bariatric centers: A national survey
Source: Clin Obes. 2021 May 24;11(4):e12461. doi: 10.1111/cob.12461 (PMC8365720; doi:10.1111/cob.12461)
Supplement: Supplementary file 1 — AppendixS1: Supporting information. [file COB-11-e12461-s001.docx]

**Supplemental data**

**Supplementary Table 1**. Survey used for identifying preoperative care in the Dutch bariatric centers

**Demographics**

- In which center are you employed?

………………..

- What is your current position?
- Surgeon
- Dietitian
- Physician assistant
- Surgical residents
- Nurse practitioner
- Other, please specify……….

**Weight loss goals**

- What is the preoperative weight loss goal?
- A patient is requested to lose weight (go to question 5)
- A patient is requested to maintain weight or lose weight (go to question 5)
- Patient’s weight does not matter, may even gain weight
- Why do preoperative weight changes not matter?

……………………………………..

- Are you aiming for a specific number of kilograms/ or a specific percentage total weight loss preoperatively?
- Yes, … kg and/or …%
- No
- I do not know
- Will surgery be postponed if the desired weight is not achieved?
- Yes
- No

**Dietary recommendation**

- Do you recommend a diet preoperatively?
- Yes
- Only on indication
- No (go to question 28)
- Does the recommended diet depend on the type of surgery? (for example gastric bypass or gastric sleeve)
- Yes
- No (go to question 10)
- I do not know (go to question 10)
- Why is the recommended diet different depending on the type of surgery? What is the difference?

…………………………………………..

- Does the recommended diet depend on the body weight/BMI of the patient?
- Yes
- No (go to question 13)
- I do not know (go to question 13)
- Why is the diet different depending on the patient’s body weight/BMI? What is the difference?

………………………………………..

- What is the cutoff point in terms of body weight/BMI?

………………………………………..

- Is the recommended diet different in patients with Diabetes Mellitus?
- Yes
- No
- I do not know
- Is the recommended diet different in patients with other additional comorbidities (for example NASH, liver failure, kidney failure)?
- Yes
- No (go to question 16)
- I do not know (go to question 16)
- What are these comorbidities? What is different in the recommended diet if these comorbidities are present?

…………………………………………..

- What is the diet consistency?
- Liquid meal replacements/shakes (e.g. Modifast, Optifast)
- Adjusted quantity of regular food products
- Combination of meal replacements/shakes and regular food products
- Other, please specify……….
- In case of a liquid meal replacement diet consistency, which food products are patients allowed to consume next to the diet? Multiple answers possible
- Raw vegetables
- Boiled/steamed vegetables
- Clear soups or bouillon
- Dairy products
- Other, please specify……….
- No food products allowed next to the meal replacements
- What is the energy value of the preoperative diet?
- Kcal per day ………..
- Individual advice
- I do not know
- What is the diet composition of the preoperative diet? Multiple answers possible
- Grams of protein per day: ….
- Grams of carbohydrates per day: ….
- Grams of fat per day: …..
- Individual advice
- I do not know
- What is the duration of the preoperative diet?
- Slider 1 -6 weeks (one decimal)
- Individual advice
- Wat is the recommendation regarding eating moments per day in the preoperative diet?
- Slider 1-10
- Individual advice
- Do you recommend a fluid recommendation next to the preoperative diet?
- Yes
- No (go to question 25)
- I do not know (go to question 25)
- If yes, what is the amount of fluid recommended per day?

Amount of liters:….

- If yes, what type of fluid do you recommend next to the preoperative diet? Multiple answers possible
- Water
- Coffee and tea without milk or sugar
- Soda without sugar
- Clear soups or bouillon
- Other, please specify……….
- I do not know
- What is the goal of the preoperative diet? Multiple answers possible.

Rank the chosen answers from most importance at the top to least importance as the lower.

- Weight loss
- Liver volume reduction
- Decrease surgical complexity
- Decrease complication rate
- Patients’ behavioral change
- Prepare patient and getting used to post surgery eating habits
- Other, please specify……….
- The recommended preoperative diet is based on: more than one answers possible
- Guideline
- Scientific evidence
- Clinical experience
- Other, please specify……….
- Please give us an indication of how compliant patients are with the recommended preoperative diet?
- >75% of patients follow the recommended diet
- 50-75% of patients follow the recommended diet
- <50% of patients follow the recommended diet

**Nutritional supplement recommendations**

- Do you recommend nutritional supplements in the preoperative phase?
- Yes
- No (go to question 44)
- I do not know (go to question 44)
- Do you recommend protein supplementation preoperatively?
- Yes
- No (go to question 32)
- Only on indication (go to question 31)
- If yes, what dose is recommended? When does a patient need to start with protein supplements? Please describe shortly your motivation to recommend protein supplements? (go to question 32)

………………..

- If only on indication, what are the indications to recommend protein supplements? What dose is recommended? When does a patient need to start with protein supplements?

……………….

- Do you recommend multivitamin supplements preoperatively?
- Yes
- No (go to question 35)
- Only on indication (go to question 34)
- If yes, what dose is recommended? When does a patient need to start with multivitamin supplements? (go to question 35)

…………………

- If only on indication, what are the indications to recommend multivitamin supplements? What dose is recommended? When does a patient need to start with multivitamin supplements?

……………….

- Do you recommend calcium and/or vitamin D supplements preoperatively?
- Yes, only calcium
- Yes, only vitamin D
- Yes, both calcium and vitamin D
- Only on indication (go to question 37)
- No (go to question 38)
- If yes, when does a patient need to start with calcium and/or vitamin D supplements? (go to question 38)

…………………..

- If only on indication, what are the indications to recommend calcium and/or vitamin D supplements? When does a patient need to start with calcium and/or vitamin D supplements?

……………………

- Do you recommend probiotics preoperatively?
- Yes
- No (go to question 41)
- Only on indication (go to question 40)
- If yes, when does a patient need to start with taking probiotics? Please give a short description of your motivation to recommend probiotics. (go to question 41)

………………………………

- If only on indication, what are the indications to recommend probiotics? When does a patient need to start taking probiotics?

…………………………

- Do you recommend any other supplement preoperatively? (e.g. omega-3 fatty acids, weight loss pills)
- Yes
- No (go to question 44)
- Only on indication
- If yes, what is the product you recommend? Please give a short description of your motivation to recommend this product.

……………………

- If only on indication, what are these indications? What is the product you recommend? Please give a short description of your motivation to recommend this product.

………………………..

**Physical activity recommendation**

- Are patients given a recommendation regarding their activity (low intensity) level preoperatively?
- Yes (go to question 46)
- No (go to question 48)
- Only on indication
- If only on indication, what are the indications to recommend physical activity (low intensity)?

………………

- If yes and only on indication, is it just recommendation or physical activity under supervision? Please give a short description of the organization of this supervision.

………………..

- If yes and only on indication, what type of physical activity (low intensity) is recommended, and at what frequency, intensity and duration?

……………….

- Are patients recommended (to start with) physical activity (mod- to high-intensity) preoperatively?
- Yes (go to question 50)
- No (go to question 52, except if question 44 was ‘no’ as well go to question 53)
- Only on indication
- If only on indication, what are the indications to recommend physical activity (mod-to high-intensity)?

………………

- If yes and only on indication, is it only recommended or physical activity under supervision? Please give a short description of the organization of this supervision.

………………..

- If yes and only on indication, what type of physical activity (mod- to high-intensity) is recommended, and at what frequency, intensity and duration?

………………

- What is the goal of the preoperative physical activity recommendation? Multiple answers possible.

Rank the chosen answers on top of most importance to lower importance.

- Weight loss
- Liver volume reduction
- Decrease surgical complexity
- Decrease complication rate
- Behavioral change in the patient
- Improve physical fitness
- Other, please specify……….
- How long have you been using the recommendations described in this survey related to weight loss goal, and dietary, nutrition supplement, and physical activity recommendations on average?
- Less than 1 year
- More than 1 year
- More than 3 years
- More than 5 years
- Other, please specify……….
- I do not know
- Do you have additional comments, questions, or suggestions?

………………………

- Would you like to receive the results of this survey? Please fill in your email address. This will only be used to send the results of this survey. Your answers to this survey will remain anonymous.

……………………..

**Supplementary Figure 1.** Underlying reasons for preoperative dieting in the eighteen bariatric centers.
